# Better late than never: sleep still supports memory consolidation after prolonged periods of wakefulness

**DOI:** 10.1101/lm.053660.122

**Published:** 2023-09

**Authors:** Marit Petzka, Ondrej Zika, Bernhard P. Staresina, Scott A. Cairney

**Affiliations:** 1 Max Planck Research Group NeuroCode, Max Planck Institute for Human Development, 14195 Berlin, Germany; 2 Max Planck University College London Centre for Computational Psychiatry and Aging Research, 14195 Berlin, Germany; 3 Institute of Psychology, University of Hamburg, 20146 Hamburg, Germany; 4 Department of Experimental Psychology, University of Oxford, Oxford OX2 6GG, United Kingdom; 5 Oxford Centre for Human Brain Activity, Wellcome Centre for Integrative Neuroimaging, Department of Psychiatry, University of Oxford, Oxford OX3 9DU, United Kingdom; 6 Department of Psychology, University of York, York YO10 5DD, United Kingdom; 7 York Biomedical Research Institute, University of York, York YO10 5DD, United Kingdom

## Abstract

While the benefits of sleep for associative memory are well established, it is unclear whether single-item memories profit from overnight consolidation to the same extent. We addressed this question in a preregistered, online study and also investigated how the temporal proximity between learning and sleep influences overnight retention. Sleep relative to wakefulness improved retention of item and associative memories to similar extents irrespective of whether sleep occurred soon after learning or following a prolonged waking interval. Our findings highlight the far-reaching influences of sleep on memory that can arise even after substantial periods of wakefulness.

Imagine a time when you walked along the coastline; you might have passed by a lighthouse or heard seagulls cry. Remembering this episode depends on its successful consolidation; that is, its transformation from a new experience into a stable memory trace. A large body of research has established the relevance of sleep for this transformation process, such that memory consolidation is superior after sleep relative to an equivalent period of wakefulness (defined as sleep-associated consolidation) (Jenkins and Dallenbach 1924; Diekelmann and Born 2010; Rasch and Born 2013).

Remembering episodes requires memories for single elements (e.g., the lighthouse in the above example) as well as memories for relations between them (e.g., link between the lighthouse and the coastline). Previous research has shown that the retrieval of memories for single elements (i.e., item memories) and memories for relations (i.e., associative memories) is associated with distinct patterns of brain activity (Davachi 2006; Eichenbaum et al. 2007; Mayes et al. 2007; but see Squire et al. 2007). While associative memory retrieval is associated with activity in the hippocampus, item memory is predominantly associated with activity in extrahippocampal regions (Davachi et al. 2003; Kirwan and Stark 2004; Ranganath et al. 2004). Since extant models of overnight memory consolidation have typically emphasized the role of sleep for hippocampus-dependent memories (Born and Wilhelm 2012; Rasch and Born 2013), we tested whether associative memories benefit more from sleep than item memories. Thus, we conducted a well-powered and preregistered online study^1^ that directly compared the effects of sleep on item and associative memory.

Studies investigating overnight memory processing typically place sleep in close proximity to the learning episode. This follows from an assumption that the magnitude of any sleep-associated memory benefit depends on the time elapsed between encoding and sleep, with shorter intervals resulting in better retention. Indeed, previous work has shown that, when memory is assessed 24 h after encoding, individuals who sleep within a few hours of learning perform better than those who sleep after a more substantial waking interval of 12 h (Gais et al. 2006; Talamini et al. 2008). The purported time-dependent nature of sleep-associated consolidation is inherent to many experimental designs in the sleep and memory literature, meaning that the interval between encoding and sleep is typically kept as short as possible (Ngo et al. 2013; Schreiner and Rasch 2015; Schönauer et al. 2017; Cairney et al. 2018; Petzka et al. 2022). To investigate how the timing of sleep influences the consolidation of item and associative memory, we further assessed retention performance after an overnight sleep opportunity that occurred either soon after encoding or following an initial 12 h of daytime wakefulness.

We used a paradigm that permitted the assessment of item and associative memory with comparable measures. Additionally, we manipulated (1) whether encoding was followed by overnight sleep or daytime wakefulness (interval: sleep vs. wake) and (2) the duration of the interval between learning and retrieval (duration: 12 h vs. 24 h). This resulted in a two × two between-subjects design with four groups (assigned randomly): 12-h sleep (*n* = 40), 12-h wake (*n* = 37), 24-h sleep–wake (*n* = 40), and 24-h wake–sleep (*n* = 35) (Fig. 1A).

**Figure 1.**
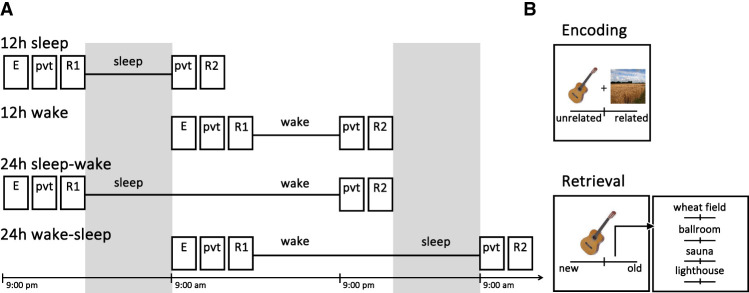
Experimental procedures and task. (*A*) Participants were randomly assigned to one of four groups (12-h sleep, 12-h wake, 24-h sleep–wake, or 24-h wake–sleep). The 12-h sleep and 24-h sleep–wake groups completed encoding (E) and the preinterval retrieval test (R1) in the evening between 7:00 p.m. and 10:00 p.m. The postinterval retrieval test (R2) was completed 12 h later (between 7:00 a.m. and 10:00 a.m.) by the 12-h sleep group and 24 h later (between 7:00 p.m. and 10:00 p.m.) by the 24-h sleep–wake group. The 12-h wake and 24-h wake–sleep groups completed encoding and the preinterval retrieval test in the morning between 7:00 a.m. and 10:00 a.m. The postinterval retrieval test was completed 12 h later (between 7:00 p.m. and 10:00 p.m.) by the 12-h wake group and 24 h later (between 7:00 a.m. and 10:00 a.m.) by the 24-h wake–sleep group. A short psychomotor vigilance task (PVT) was completed before each retrieval test (see the Supplemental Material for PVT results). (*B*) During encoding, 100 object–scene pairs were presented (one after another) and participants rated their semantic relatedness using a slider (scale 0–100). At retrieval, half of the encoded objects and 50 unseen objects (lures) were presented in isolation (one after another). Participants indicated whether the object was old or new using a slider (to indicate their confidence; scale 0–100) (see the Supplemental Material for results on confidence ratings). For objects classified as old (slider response ≥ 50), participants also indicated which scene the object had been presented with at encoding (rating their confidence with a slider; scale 0–100).

Our sample size was estimated by applying a safeguard power analysis (Perugini et al. 2014) with effect sizes obtained from a previous study using a similar experimental design (Talamini et al. 2008). Instead of calculating the sample size based on the reported effect sizes, the safeguard power analysis used the lower bound of the confidence intervals around the reported effect sizes (avoiding overestimation of effect sizes and thus underestimation of required sample sizes) (Perugini et al. 2014). The first effect corresponded to superior memory performance after a 12-h interval containing overnight sleep (vs. 12 h of daytime wakefulness; Cohen's *d* = 0.89, 80% CI = 0.60). The second effect corresponded to superior memory performance after a 24-h interval when overnight sleep occurred during the first 12 h after encoding (vs. sleep during the second 12 h; Cohen's *d* = 0.87, 80% CI = 0.58). With an alpha level of 0.05 and a power of 0.80, the required sample sizes across all experimental groups were *N* = 144 and *N* = 152 for the first and second effect sizes, respectively.

Overall, 198 participants were recruited via the participant recruitment system of the University of Birmingham. Thirty participants did not return for the postinterval session, 11 participants were excluded because they reported that they took a nap during the day, and five participants were excluded based on their memory performance on the preinterval retrieval test (Supplemental Table S1). This resulted in a final sample of *N* = 152 participants (female = 129, nonbinary = 4, male = 19, mean ± SD age = 20.52 yr ± 5.12 yr). The study was approved by the University of Birmingham Research Ethics and Governance Committee, and participants were reimbursed with experimental participation credits (University of Birmingham Psychology students).

The experiment consisted of an encoding phase, a psychomotor vigilance task (see the Supplemental Material), and a retrieval phase. Before starting the experiment proper, participants completed short practice versions of all phases. Each phase was implemented with JavaScript and the jsPsych toolbox (de Leeuw 2015) and was hosted on an online experiment server (https://www.cognition.run). Participants performed the experiment remotely. To ensure that they carried out the sessions at the correct time (between 7:00 a.m./p.m. and 10:00 a.m./p.m.), participants could only open the HTML links during the allotted time window. Before they received the HTML link, they were instructed to not drink alcohol the day before the first testing session or between both testing sessions and to follow their normal sleep pattern. To increase adherence, participants could flexibly decide which day they would take part in the study. At the end of the experiment, participants estimated their sleep duration during the previous night (mean ± 95% CI across all three conditions containing sleep = 7.72 h ± 0.23 h; for each condition individually: 12-h sleep: 7.30 h ± 0.47 h; 24-h sleep–wake: 7.96 h ± 0.42 h; 24-h wake–sleep: 7.94 h ± 0.31 h; *F*_(2,110)_ = 3.42, *P* = 0.036) and indicated whether they had taken a nap.

At encoding, participants were presented with 100 object–scene pairs. Each trial began with a fixation cross (1.5 sec) followed by a randomly selected object and scene presented side by side. Participants were instructed to memorize the object–scene pair and rate its semantic relatedness using a slider (scale ranging from 0 to 100; furthest left [0] = not related at all, furthest right [100] = highly related) (Fig. 1B). Upon releasing the slider, the next trial started. Objects were trial-unique, but scenes were repeatedly selected out of four possible images (lighthouse, sauna, ballroom, and wheat field), which were equally distributed across objects. Four repeated scenes were chosen to enable a four-alternative forced-choice retrieval test (Staresina et al. 2012).

For the preinterval retrieval test (four-alternative forced choice), participants were presented with half (*n* = 50) of the encoded objects and 50 unseen objects (lures). Each retrieval trial began with a fixation cross (1.5 sec) followed by a randomly selected object/lure. Participants were instructed to rate how confident they were that the object was old (i.e., presented at encoding) or new (i.e., not presented at encoding) using a slider to obtain a fine-grained measure of confidence (scale ranging from 0 to 100; furthest left [0] = definitely new, furthest right [100] = definitely old). If they indicated that the object was old (slider response ≥50), they were asked to indicate the scene that the object had been presented with at encoding. Each scene label was shown with a corresponding slider (scale = 0–100); participants chose one of the four scene labels by moving the relevant slider to a position that indicated the confidence in their choice (furthest left [0] = completely unsure, furthest right [100] = completely sure). Once a scene label/slider was chosen, the other scene labels/sliders became inactive. The order of the scene labels/sliders was randomly shuffled across trials. On releasing the chosen slider, the next trial started. If participants indicated that the object was new (slider response <50), scene labels/sliders were still presented (for 5 sec) but were inactive from the beginning. Hence, indicating that an object was new did not result in finishing the task any faster. Retrieval performance for the object and scene elements of the task provided metrics of item and associative memory, respectively.

The postinterval retrieval test (four-alternative forced choice) followed procedures identical to those of the preinterval retrieval test, with the exception that the other half of the learned objects (*n* = 50) and a new set of 50 lures were presented in random order. Assessing memory for different sets of object–scene pairs in the preinterval and postinterval retrieval tests ensured that any effect of sleep on retention was not influenced by retrieval practice effects (Roediger and Karpicke 2006; Roediger and Butler 2011; Bäuml et al. 2014; Antony et al. 2017).

To measure item memory performance, we calculated *d*′. Because *d*′ cannot be estimated with extreme hit (*h* = 0 or *h* = 1) or false alarm rates (*z* = 0 or *z* = 1), a log linear approach was adapted to safeguard our analysis against such values (Hautus 1995). Specifically, 0.5 was added to each cell in the obtained contingency table representing the frequencies of hits, misses, false alarms, and correct rejections.

To permit a direct comparison of the effect of sleep on item and associative memory, associative memory performance was also assessed with *d*′ (Supplemental Table S1). Because we used a four-alternative forced-choice task at retrieval, *d*′ could be estimated here together with bias for each option. The signal detection model for two choices (e.g., old vs. new) could be extended to *m*-alternative forced choices as outlined by DeCarlo (2012). By applying an hierarchical Bayesian estimation approach, all parameters (*d*′ and the bias parameters *b1*, *b2*, *b3*) were simultaneously estimated. Separate hyperpriors for each session and experimental group were used. This approach allows for meaningful partial pooling across participants within each group and session, which has been shown to improve statistical inference (Valton et al. 2020). For a detailed specification of the model, please see the Supplemental Material.

For both item and associative memory, retention was then calculated as *d*′_retention_ = *d*′_postinterval_ − *d*′_preinterval_ (see Supplemental Table S2 for hit and false alarm rates and accuracies for preinterval and postinterval sessions). Difference scores were used to account for marginal differences between conditions (12-h sleep, 12-h wake, 24-h sleep–wake, and 24-h wake–sleep) at preinterval retrieval (item memory: *F*_(3,148)_ = 0.31, *P* = 0.817; associative memory: *F*_(3,148)_ = 2.62, *P* = 0.053). We ran a linear model with *d*′_retention_ as the dependent measure and interval (sleep vs. wake), duration (12 h vs. 24 h), and memory type (item vs. associative memory) as fixed effects.

We hypothesized that prompt, postlearning sleep, as compared with wakefulness, leads to superior memory performance irrespective of whether retrieval takes place 12 h or 24 h after encoding (i.e., 12-h sleep > 12-h wake and 24-h sleep–wake > 24-h wake–sleep). Intriguingly, however, our results revealed a different pattern (interval × duration: *F*_(1,296)_ = 10.93, *P* = 0.001). Whereas memory retention was significantly higher in the 12-h sleep than in the 12-h wake condition (*t*_(149.51)_ = 2.60, *P* = 0.010), there was no difference in performance between the 24-h sleep–wake and 24-h wake–sleep conditions (*t*_(147.98)_ = −0.85, *P* = 0.395), suggesting that the memory benefits of overnight consolidation were not contingent on sleep occurring soon after learning (Fig. 2A). Furthermore, we did not find a significant difference between the 12-h wake condition and 24-h sleep–wake condition (*t*_(150.75)_ = 0.70, *P* = 0.482) or between the 12-h wake condition and 24-h wake–sleep condition (*t*_(141.13)_ = −0.11, *P* = 0.915).

**Figure 2.**
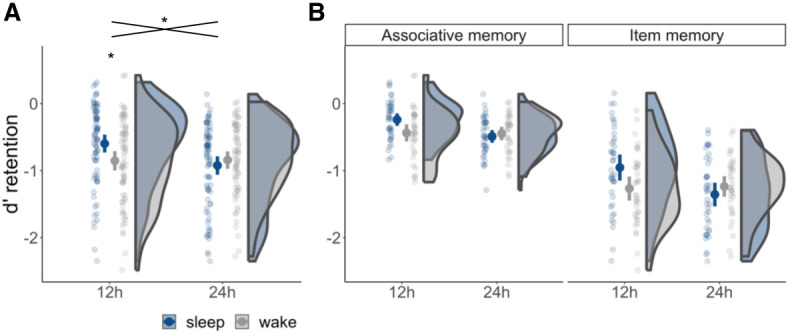
Overnight consolidation is not contingent on sleep taking place soon after learning. (*A*) Memory retention after a 12-h interval was significantly higher among participants who had slept (12-h sleep condition) relative to those who had remained awake (12-h wake condition). In contrast, memory retention after a 24-h interval did not differ between participants who slept in the first few hours after learning (24-h sleep–wake; shown in blue at 24 h) and those who slept after an initial 12-h waking interval (24-h wake–sleep; shown in gray at 24 h). Data are shown for item and associative memory combined. (*) *P* < 0.05. (*B*) The pattern presented in *A* did not significantly differ between item and associative memory. Figures include density plots, group means with 95% confidence intervals, and single-participant data (averaged over trials).

Contrary to our hypothesis that the benefit of sleep would be amplified for associative memory relative to item memory, the foregoing pattern of results was highly comparable across memory types (interval × duration × memory type: *F*_(1,296)_ = 0.90, *P* = 0.343) (Fig. 2B). A complementary Bayesian analysis (Cauchy distribution with 0, 0.707 as prior distribution) conducted on the three-way interaction [item memory (interval × duration) = associative memory (interval × duration)] provided substantial evidence for the null (BF_01_ = 5.53) (Jarosz and Wiley 2014). No other interaction containing the factor memory type was significant (*P* > 0.343).

Because we formulated individual hypotheses for item and associative memory in our preregistration, we conducted two linear models separately for each memory type with *d*′_retention_ as the dependent measure and interval (sleep vs. wake) and duration (12 h vs. 24 h) as fixed effects. A significant interval × duration interaction was observed in both models (item memory: *F*_(1,148)_ = 6.05, *P* = 0.015; associative memory: *F*_(1,148)_ = 5.50, *P* = 0.020), matching the results of our main linear model.

Our observation that sleep improves the retention of both item and associative memories is in keeping with a recent adaptation to the influential active systems model of sleep-associated memory processing, which accounts for memories that are classified as both hippocampus-dependent and non-hippocampus-dependent (Klinzing et al. 2019). Supported by recent work in animals (Sawangjit et al. 2018; Kim et al. 2023) and humans (King et al. 2017), hippocampal memory reactivation during slow-wave sleep is proposed to capture nonhippocampal components of prior experience and, in doing so, drive the consolidation of memory traces that are seemingly hippocampus-independent. Hence, even for memories that do not rely on the hippocampus at encoding, overnight hippocampal engagement might represent a domain-general mechanism underpinning the formation of long-term memory. An important point in the context of our current results, however, is that encoding of item memories relies on both hippocampal and extrahippocampal areas (Davachi et al. 2003; Kirwan and Stark 2004; Ranganath et al. 2004; for review, see Davachi 2006). Whether processes underlying non-hippocampus-dependent memory consolidation also support item memory consolidation is currently unclear.

We observed a sleep-associated consolidation effect after 12 h (12-h sleep > 12-h wake). We then tested whether this effect is time-dependent; that is, contingent on sleep occurring soon after learning. Contrary to our hypothesis, we did not observe superior memory consolidation when sleep followed soon after learning (24-h sleep–wake ∼ 24-h wake–sleep). The indication of a similar sleep-associated consolidation effect among individuals who remained awake for the first 12 h after learning (compared with individuals sleeping directly after learning and then staying awake) raises questions about the role that prior wakefulness plays in overnight memory processing. Hippocampal–cortical interactions have been observed during wakeful rest following associative memory encoding, with the magnitude of such interactions predicting individual differences in later retrieval (Tambini et al. 2010). This raises the possibility that memory reactivations during wakefulness support consolidation processes emerging in subsequent sleep. Indeed, more recent work has shown that memory reactivation in the hippocampus during awake rest predicts later retrieval performance but only when the intervening period contains a night of sleep (Schapiro et al. 2018). When the intervening period was filled with wakefulness, retrieval performance in the long run could not be predicted by memory reactivation during awake rest. Further evidence supporting the notion that reactivation during wakefulness requires subsequent sleep for long-lasting memories was provided by Himmer et al. (2019). The investigators showed that systems memory consolidation (initiated during wakefulness via repeated rehearsal) required subsequent sleep to lead to long-lasting changes.

It is important to note that retrieval performance was lower in our data for participants who remained awake in the 12-h condition (12-h wake) as compared with those who slept (12-h sleep). Together with the previous literature, this suggests that while hippocampal memory reactivation during wakefulness might relate to performance gains across sleep, the 12-h wakefulness period in isolation does not provide the same benefit for memory consolidation as an overnight retention interval. Since we did not specifically instruct participants to rest during the 12-h wake period, these conclusions need further support from future studies, also applying neuroimaging techniques.

Future studies are additionally helpful in ruling out time of day effects and interference during wakefulness as contributing factors to our findings. For example, a nap design in which encoding and retrieval take place at approximately the same time of day across the nap/wake conditions is suitable. To reduce interference during wakefulness, a controlled distractor task could be included in the study protocol.

In sum, our findings suggest that overnight memory consolidation supports item and associative memory to similar extents (12-h sleep > 12-h wake for item and associative memory). Furthermore, the results indicate that overnight memory consolidation effects do not depend on whether sleep occurs soon after learning or following a prolonged waking interval (24-h sleep–wake vs. 24-h wake–sleep). These data suggest that the benefits of sleep for memory are widespread, reaching aspects of prior experience that differentially rely on the hippocampus (i.e., associative memory) and extrahippocampal regions (i.e., item memory). Moreover, they highlight the versatility of sleep-associated memory processing, with sleep strengthening memories that have been exposed to limited or substantial periods of wakefulness.

## Supplementary Material

Supplement 1

## Footnotes

1 Our preregistration can be found at https://osf.io/wb2zh. Deviations from our preregistered methods are highlighted in Supplemental Table S1, in which all deviations are further explained and justified.
