# Supplementary material for: Better late than never: sleep still supports memory consolidation after prolonged periods of wakefulness
Source: Learn Mem. 2023 Sep;30(9):245–9. doi: 10.1101/lm.053660.122 (PMC10547377; doi:10.1101/lm.053660.122)
Supplement: Supplement 1 [file Supplementary_material.docx]

**Supplemental Information**

**Table S1.** List of deviations from the pre-registration and their justifications.

|  | **Pre-registration** | | **Manuscript** | |
| --- | --- | --- | --- | --- |
| **1** | **Exclusion criterion – taking a nap** | | | |
|  | “All participants whose associative memory score is lower than 25% (i.e., chance level given four response options) will be excluded. “ | | “[…] Thirty participants did not return for the post-interval session, 11 participants were excluded because they reported to took a nap during the day…” | |
|  | **Justification:** The pre-registered exclusion criterion was based on associative memory performance only. However, some participants reported that they took a nap during the day (12h wake: n = 4, 24h sleep-wake: n = 3, 24h wake-sleep: n = 4). We excluded them from the main analysis. Please note, that the results do not significantly differ with or without them. | | | |
|  | **Exclusion criterion – item memory performance** | | | |
|  | “All participants whose associative memory score is lower than 25% (i.e., chance level given four response options) will be excluded. “ | “[…] and 5 participants were excluded based on their memory performance on the pre-interval retrieval test. ” | | |
|  | **Justification:** The pre-registered exclusion criterion was based on associative memory performance only. However, after applying the exclusion criterion, item memory performance for two participants was far below average (< mean – 3std). Consequently, we incorporated a second exclusion criterion (item memory performance < mean – 3std) and excluded 5 participants overall (N = 3 based on associative memory performance and N = 2 based on item memory performance). | | | |
| **2** | **d' as a measure for associative memory performance** | | | |
|  | “First (*associative memory performance*), we calculate the proportion of item hits for which the scene was correctly remembered at retrieval 1 and retrieval 2.” | | “To permit a direct comparison of the effect of sleep on item and associative memory, associative memory performance was also assessed with *d’.* ” | |
|  | **Justification:** To directly compare item and associative memory performance (independently of confidence ratings), we estimated *d’* for both. It is worth mentioning that change scores for the pre-registered method of associative memory performance are highly correlated with the change in *d’* for associative memory (r = 0.96, p < 0.001). | | | |
| **3** | **Calculation of confidence ratings** | | | |
|  | “The cumulative distribution function (CDF) of the confidence ratings for old items is calculated for retrieval 1 and 2. Analogous to the Kolmogorov-Smirnov (KS) test, the maximal distance between the CDF of retrieval 1 and retrieval 2 is identified. The maximal distance reflects the largest difference in frequency across all bins. […] The maximal distance is used as the dependent variable. ” | | | “We therefore averaged confidence ratings at both the pre-interval and post-interval retrieval session, separately for objects and scenes.” |
|  | **Justification:** We pre-registered to calculate the change in confidence based on cumulative distribution functions. However, as the results did not significantly differ compared to simply averaging confidence ratings, we decided to report the more parsimonious approach, i.e., the averaged confidence ratings. | | | |

*Psychomotor vigilance task*

Participants completed a psychomotor vigilance task (PVT) directly before the pre-interval and post-interval retrieval tasks. The PVT began with a black fixation cross, which was presented in the centre of the screen. After 4 s (jitter ± 2 s), the fixation cross turned red and participants were instructed to press the space bar as quickly as possible. On each keypress, feedback about participants’ reaction time was provided. The PVT included 25 trials.

We calculated two dependent variables: 1) averaged reaction time across trials with a reaction time < 500ms, and 2) frequency of attentional lapses (i.e. the number of trials with reaction time > 500ms; Basner & Dinges, 2011). Both measures of the pre- and post-interval session (PVT*_pre-interval_,* PVT*_post-interval_*) were considered as well as the change between sessions (PVT*_change_* = PVT*_post-interval_* – PVT*_pre-interval_*). All variables (averaged reaction time and attentional lapses for PVT*_pre-interval,_* PVT*_post-interval_* and PVT_change_) were applied to linear models with *condition* as the predictor. None of the six models were statistically significant (all p > .061, uncorrected), suggesting that the results of our main analysis did not arise from between-condition differences in participant vigilance.

*Computational model*

The model estimates latent parameters *d’, b1, b2,* and *b3* from choices ***y*** made by the participant ***p*** and the objective signal ***X***. It assumes that a response ***y*** for each alternative ***m*** on trial ***t*** within session ***s*** for participant ***p*** is given by a Bernoulli likelihood $y_{\left( p,s,m,t \right)}\sim Bernoulli\left( q_{\left( p,s,m,t \right)} \right)$

where ***q*** represents the probability of each choice according to Signal Detection Theory (for details see DeCarlo, 2012) as follows:

$$q_{1}=\Phi\left( b_{1}-b_{2}+dZ+\epsilon_{1} \right)*\Phi\left( b_{1}-b_{3}+dZ_{a}+\epsilon_{1} \right)*\Phi\left( b_{1}-dZ_{1}+\epsilon_{1} \right)$$

$$q_{2}=\Phi\left( b_{2}-b_{1}+dZ+\epsilon_{2} \right)*\Phi\left( b_{2}-b_{3}+dZ_{b}+\epsilon_{2} \right)*\Phi\left( b_{2}-dZ_{2}+\epsilon_{2} \right)$$

$$q_{3}=\Phi\left( b_{3}-b_{1}+dZ_{a}+\epsilon_{3} \right)*\Phi\left( b_{3}-b_{2}+dZ_{b}+\epsilon_{3} \right)*\Phi\left( b_{3}-dZ_{3}+\epsilon_{3} \right)$$

$$Z=X_{1}-X_{2}$$

$$Z_{a}=X_{1}-X_{3}$$

$$Z_{b}=X_{2}-X_{3}$$

$$Z_{1}=1-2X_{1}-X_{2}-X_{3}$$

$$Z_{2}=1-X_{1}-2X_{2}-X_{3}$$

$$Z_{3}=1-X_{1}-X_{2}-2X_{3}$$

***X_m_*** represents the correct class ***m*** on each trial ***t****. m = 4 does not have to be defined since it can be derived from X_1_, X_2_, X_3._*

All five parameters (*d’*, *b1*, *b2*, *b3* and $\epsilon$) follow identical hierarchical structure, therefore here we only outline parameter ***d’*** for conciseness. The value of the individual parameter ***d’*** for each session and participant is assumed to come from a hierarchical Normal distribution with separate mean and standard deviation for each combination of session ***s*** (1,2) and group ***g*** (12h-sleep, 12h-wake, 24hr-sleep-wake, 24hr-wake-sleep). For each group/session combination we assume a hyperprior with mean ***D*** and standard deviation $\sigma$ with weakly informative priors:

$$D_{\left( g,s \right)}\sim Normal\left( 0, 10 \right)$$

$$\sigma_{\left( g,s \right)}\sim HalfCauchy\left( 0, 2.5 \right)$$

The value of ***d’*** for each session and participant is then given by

$$d_{\left( p,s \right)}\sim Normal\left( D_{\left( g,s \right)}, \sigma_{\left( g,s \right)} \right)$$

The model was specified and estimated using R (version 4.1) and the rstan package (version 2.21.7). Markov Chain Monte Carlo sampling was performed using the No U-Turn Sampler (NUTS) across 32 chains with 350 samples each (200 warmup).

**Table S2.** Pre- and post-interval item memory (hits, misses, false alarms, correct rejections; maximum number of trials = 50) and associative memory (hits; maximum number of possible associative hits = item hits) (mean ± 95% CIs).

|  | Item memory | | | | Associative memory | |
| --- | --- | --- | --- | --- | --- | --- |
|  | Hits | Misses | False Alarms | Correct rejections | | Hits |
| pre-interval |  |  |  |  | |  |
|  |  |  |  |  | |  |
| 12h sleep | 43.83±2.08 | 6.18±2.08 | 2.10±0.87 | 47.90±0.87 | | 31.98±3.14 |
| 12h wake | 44.08±1.60 | 5.92±1.60 | 1.14±0.55 | 48.86±0.55 | | 35.22±2.71 |
| 24h sleep-wake | 43.48±1.93 | 6.50±1.93 | 1.45±0.54 | 48.55±0.54 | | 32.18±2.98 |
| 24h wake-sleep | 42.76±1.76 | 7.24±1.76 | 2.62±1.79 | 47.38±1.79 | | 30.46±2.90 |
|  |  |  |  |  | |  |
| post-interval |  |  |  |  | |  |
| 12h sleep | 33.60±3.30 | 16.38±3.30 | 3.60±1.59 | 46.40±1.59 | | 21.53±3.06 |
| 12h wake | 31.62±3.38 | 18.38±3.38 | 3.38±1.23 | 46.62±1.23 | | 19.38±3.44 |
| 24h sleep-wake | 32.70±2.75 | 17.28±2.75 | 5.08±1.15 | 44.93±1.15 | | 18.08±2.85 |
| 24h wake-sleep | 29.81±2.58 | 20.18±2.58 | 3.95±1.91 | 46.05±1.91 | | 15.73±2.29 |

*Confidence ratings*

Alongside memory performance (*d’*), we pre-registered to analyse confidence ratings as an index of memory strength. We therefore averaged confidence ratings at both the pre-interval and post-interval retrieval session, separately for objects and scenes (thus providing separate confidence indices for item and associative memory; Table S1.4). Both measures were then subtracted between sessions (confidence*_change_* = confidence*_post-interval_* – confidence*_pre-interval_*) and applied to a linear mixed effects model with *interval* (sleep vs. wake), *duration* (12 h vs. 24 h) and *memory type* (item vs. associative memory) as fixed effects. Confidence was significantly lower after 24 h than 12 h (main effect *duration*: F(1, 281) = 10.84, p = .001), and lower for item memory relative to associative memory (main effect *memory type*: F(1, 281) = 12.30, p < .001). However, confidence did not differ between sleep and wake groups (main effect *interval*: F(1, 281) = 0, p < .988), nor was any interaction including *interval* significant (all p > .067).
